# Exploring the risk factors for ischemic cerebrovascular disease in systemic lupus erythematosus: A single-center case-control study

**DOI:** 10.3389/fimmu.2022.978910

**Published:** 2022-09-27

**Authors:** Li Su, Zhigang Qi, Shaochen Guan, Lian Wei, Yi Zhao

**Affiliations:** ^1^ Department of Rheumatology and Allergy, Xuanwu Hospital, Capital Medical University, Beijing, China; ^2^ Department of Radiology and Nuclear Medicine, Xuanwu Hospital, Capital Medical University, Beijing, China; ^3^ Evidence-Based Medical Center, Xuanwu Hospital, Capital Medical University, Beijing, China

**Keywords:** systemic lupus erythematosus, ischemic cerebrovascular disease, stroke, intracranial arterial stenosis, risk factors

## Abstract

**Objectives:**

Ischemic cerebrovascular disease (ICVD) is one of the most common and severe complications in systemic lupus erythematosus (SLE). We aim to explore the risk factors for ICVD in SLE and to assess their associated clinical characteristics.

**Methods:**

In this study, 44 lupus patients with ICVD (ICVD-SLE) and 80 age- and sex-matched lupus patients without ICVD (non-ICVD-SLE) who were hospitalized in our center between 2014 and 2021 were enrolled. A comprehensive set of clinical and socio-demographic data was recorded. In the ICVD-SLE group, the modified Rankin score (mRS) at 90 days after the occurrence of ICVD, the brain MRI, and arterial ultrasonography findings were collected. Group comparisons were made with continuous variables using an independent t-test or the Mann–Whitney test, and with categorical variables using the chi-square test or Fisher exact test. Multivariate logistic regression analysis was performed to identify the risk factors for ICVD in SLE. Patients with ICVD-SLE were divided into three subgroups according to the gradations of intracranial arterial stenosis (ICAS). The subgroup comparisons were performed by one-way ANOVA test or Kruskal–Wallis test.

**Results:**

Of the 44 patients with ICVD, 45% had a large-vessel ischemic stroke, 50% had a symptomatic lacunar stroke, and 9% had a transient ischemic attack. 2 (4.5%) had both large-vessel ischemic stroke and symptomatic lacunar stroke. Multivariate logistic regression analysis showed that cutaneous vasculitis (OR=7.36, 95% CI=2.11–25.65), anticardiolipin antibody (aCL) (OR=4.38, 95% CI=1.435–13.350), and lupus anticoagulant (LA) (OR=7.543,95% CI=1.789–31.808) were the risk factors, and hydroxychloroquine (HCQ) therapy (OR=0.198, 95% CI=0.078–0.502) was the protective factor, after controlling for confounders. During the analysis of the subgroups, no significant difference was observed between the patients in the group without internal carotid arterial occlusion (ICAS) and those with severe ICAS except for diagnostic delay. However, patients in the moderate ICAS group were older when SLE occurred (P<0.01), had a longer diagnostic delay (P<0.01), a lower percentage of hypocomplementemia (P=0.05) and steroids and HCQ therapy (P=0.01, P=0.05, respectively), a trend toward lower mRS score, but a higher incidence of carotid atherosclerotic plaque (P<0.01), when compared with the other two subgroups.

**Conclusion:**

Cutaneous vasculitis and antiphospholipid antibodies (aPLs) are associated with an increased risk of ICVD, while HCQ therapy may provide protection against ICVD in SLE. The ICVD in younger lupus patients is associated with complement-mediated inflammation and poorer outcome, and require immunosuppressive therapy, whereas the ICVD in elderly patients are characterized by moderate ICAS and carotid atherosclerotic plaques.

## Introduction

Systemic lupus erythematosus (SLE) is a chronic systemic autoimmune disease (1, 2). Ischemic cerebrovascular disease (ICVD) is one of the most common and severe complications of SLE. Its prevalence varies from 3% to 20% and accounts for up to 15% of deaths associated with SLE (3). It is one of the leading causes of increased morbidity and mortality in SLE. ICVD mainly includes ischemic stroke and transient ischemic attacks (TIA) (4), which are primarily caused by large-vessel disease, small-vessel disease, embolism, systemic hypoperfusion, and coagulation disorders. Cerebral small-vessel disease (SVD) (5) can cause a TIA, lacunar infarction, or minor stroke, with or without clinical symptoms (6–8). Patients with SLE are at higher risk (>200%) of developing ischemic stroke than are controls without SLE from the general population, and this risk further increased in women and younger populations (<50 years of age), particularly in the first year following diagnosis (1). In the study by Tsoi et al., border zone infarcts and infarcts in multiple regions on imaging were significantly more prevalent in SLE patients when compared to non-SLE stroke patients (9). There are several pathological hypotheses for ICVD in SLE, which includes lupus-related hypercoagulable state (the presence of antiphospholipid antibodies (aPLs)), proinflammatory cytokine-mediated vasculitis and vasculopathy, which could involve both large and small vessels, emboli from cardiac non-infective valvular vegetations, impaired renal function, exposure to medications like glucocorticoids (GCs), accelerated atherosclerosis, and traditional cardiovascular risk factors (e.g., hypertension, hyperlipidemia, diabetes, and smoking) (10).

However, whether the increased ICVD incidence is due to classical cardiovascular risk factors or the disease itself remains controversial (11). The disease phenotype associated with ICVD in SLE has not been fully identified. This single-center retrospective study was undertaken to evaluate the risk factors for ICVD in patients with SLE, assess the associated clinical characteristics in this unique population, and provide basis for subsequent research with larger sample sizes.

## Methods

### Patients and design

This study was a single-center case-control study. The clinical records of patients who were diagnosed with SLE by rheumatologists at Xuanwu Hospital, Capital Medical University and hospitalized between 2014 and 2021 were retrospectively reviewed. The electronic medical records database was searched by diagnostic codes; then the identified records underwent a complete chart review. To be included in the study, patients were required to be aged 18 years or older and to fulfill the 1997 American College of Rheumatology (ACR) revised criteria (12) or the ACR/Systemic Lupus International Collaborating Clinics (SLICC) 2012 criteria for SLE (13). In this study, ICVD included acute ischemic stroke and TIA (4). The diagnosis of ICVD was reviewed by a neurologist based on clinical symptoms and imaging evidence, namely, brain MRI/CT imaging and intra/extra-cranial cerebral vascular ultrasonography. Data of patients in the control group were extracted by random sampling technique from the SLE patients without ICVD, which was confirmed by a follow-up period starting at the first admission in our hospital and ending at the date of the latest medical record. Finally, 44 lupus patients with ICVD (ICVD-SLE) and 80 sex- and age-matched controls (non-ICVD-SLE) (nearly 1:2) were enrolled. The study was approved by the Ethics Committee of Xuanwu Hospital, Capital Medical University and conducted in accordance with the Declaration of Helsinki (2013 revision).

### Clinical data collection

At inclusion, clinical data were collected, including systemic manifestations, clinical activity indices, laboratory data including complements and autoantibodies, treatments, and traditional cardiovascular risk factors and socio-demographic data (sex, age at SLE onset, SLE duration, ICVD duration, and diagnostic delay). The cumulative dosages of glucocorticoid (GC) were documented through careful interviews and calculated by adding up all of the daily dosages. The dosages of intravenous and oral GC were converted to equivalent dosages of prednisone. SLE disease activity at initial presentation was assessed by the Systemic Lupus Erythematosus Disease Activity Index 2000 (SLEDAI-2K) (14). The cumulative organ damage in SLE patients was assessed by the SLICC/ACR Damage Index (SDI), which includes 12 organ systems (15). The follow-up duration was defined as the time from disease onset to the last follow-up (non-ICVD-SLE) or the first occurrence of ICVD (ICVD-SLE). In the ICVD-SLE group, the functional status of patients was assessed by the modified Rankin score (mRS) at 90 days after the occurrence of ICVD. Additionally, psychiatric symptoms and cognitive dysfunction were screened for by a psychiatrist using the following clinical screening tests: the Hamilton Depression Scale (HAMD), the Hamilton Anxiety Scale (HAMA), the Mini-Mental State Examination (MMSE), and the Montreal Cognitive Assessment (MoCA). Anti-nuclear antibody (ANA) and anti-double-stranded DNA (anti-dsDNA) were tested for by indirect immunofluorescence and enzyme-linked immunosorbent assay (ELISA). Anti-Ro/Sjogren’s syndrome A (Anti-R0/SSA), anti-La/Sjogren’s syndrome B (anti-La/SSB), anti-Smith (anti-Sm), anti-ribosomal P protein (anti-rRNP), and anti-U1 ribonucleoprotein (anti-RNP) antibodies were tested for by dot blotting. Serum titers of aPLs, including aCL and anti-β_2_GPI antibodies, were measured using standardized commercial ELISA kits. LA activity was tested for by the integrated activated partial thromboplastin time test.

### Brain magnetic resonance imaging

Brain MRI scanning was enabled by a 3.0T magnet scanner (Siemens, Erlangen, Germany) equipped with a body transmit and a 32-channel receive coil. The conventional MR sequences included T1-weighted, T2-weighted, fluid-attenuated inversion recovery (FLAIR), and diffusion-weighted imaging (DWI) sequences. T1-weighted sequence: repetition time (TR)=160 ms; echo time (TE)=3.05 ms; slice thickness=5 mm; 24 axial slices. T2-weighted sequence: TR=3,800 ms; TE=93 ms; slice thickness=5 mm; 24 axial slices. FLAIR sequence: TR=8,000 ms; TE=94 ms; slice thickness=5 mm; 24 axial slices. DWI sequence: TR=5,500 ms; TE=90 ms; slice thickness=5 mm; 24 axial slices. The MRI imaging was reviewed and assessed by a radiologist. The following eight bilateral brain regions were investigated: frontal, parietal, temporal, occipital, insular, cerebellar lobes, hippocampus, and brain stem. Abnormal MRI lesions were recorded as follows: cerebral hemorrhage, cortical infarction, subcortical infarction, lacunar infarcts, white matter hyperintensities (WMHs), cerebral microbleeds (CMBs), and brain atrophy. For WMHs, the Fazekas score was introduced to assess the severity of WMHs (5). The total Fazekas ≥3 referred to periventricular WMH Fazekas ≥3 (extending into the deep white matter) and/or deep WMH Fazekas ≥2–3 (confluent or early confluent).

### Vascular ultrasonography

The results of vascular Doppler ultrasonography (US) imaging of the carotid arteries and its major branches were reviewed. Abnormal vascular changes were recorded as follows: no stenosis (<50%), moderate stenosis (50%–69%), severe stenosis or occlusion (70%–99% or total occlusion) (16), and carotid wall thickening and plaques.

### Statistical analysis

Continuous data were presented as mean ± SD or median with interquartile range (IQR) based on whether they had normal distribution. Categorical data were presented as numbers (percentage or frequency). Patient characteristics with and without ICVD were compared with the continuous variables using independent t-test or Mann–Whitney test and the categorical variables using a chi-square test or the Fisher exact test. Given the retrospective collection of data and the observational nature of the study, some data were incomplete. Missing data were not imputed. The individuals in the control group were selected by random sampling technique to reduce the risk of selection bias. Univariate and multivariate logistic regression analysis was used to estimate odds ratios (ORs) and 95% confidence intervals (CIs) and to identify the independent risk factors for ICVD in SLE. The regression model was controlled for potential confounders (e.g., age, sex, duration, and traditional cardiovascular risk factors). In the subgroup analysis, patients in the ICVD-SLE group were divided into three subgroups, according to the gradations of intracranial arterial stenosis. Comparisons across subgroups were performed by one-way ANOVA test or Kruskal–Wallis test. A *P*-value <0.05 was considered to indicate a statistically significant difference. Statistical analyses were performed with SPSS software (IBM SPSS Statistics 25.0, Chicago, IL, USA) and Stata version 15 (Stata Corp., College Station, TX, USA).

## Results

### Demographic characteristics

All the patients in this study were ethnic Han-Chinese. The mean age at SLE onset was 38.3 ± 17.0 years (60.5% were younger than 40 years at SLE onset) with 85.5% (106/124) being female. There was no statistical difference in diagnostic delay or follow-up duration between the ICVD-SLE group and the control group (**Table 1**).

**Table 1:** Comparison of demographic, clinical, and serological characteristics between groups of lupus patients.

	ICVD-SLE					Non-ICVD-SLE	
	Total	No ICAS	Moderate ICAS	Severe ICAS	*P*-value^§^		*P*-value^‡^
	N=44	N_1_ = 20	N_2_ = 10	N_3_ = 14		N=80	
Sex (female)	36 (82%)	16 (80%)	8 (80%)	12 (86%)	1.0	70 (88%)	0.43
Age (years), mean (SD)	38.0 (26.5, 53.0)	38.0 (27.0, 49.0)	56.0 (46.0, 62.0)	27.0 (22.0, 37.0)	**<0.01**	36.5 (25.5, 53.5)	0.80
Diagnostic delay (months), median (range)^†^	3.0 (0.0, 27.5)	0.0 (0.0, 3.0)	27.5 (14.0, 46.0)	10.5 (3.0, 72.0)	**<0.01**	1.0 (0.0, 12.0)	0.21
Follow up duration (months), median (range)	69.5 (6.0, 112.0)*	87.0 (30.0, 130.0)*	21.0 (10.0, 78.0)*	64.5 (2.0, 180.0)*	0.54	44.0 (12.0, 102.0)	0.96
SLEDAI (score), median (range)	10.0 (7.0, 13.0)	10.0 (8.0, 13.5)	10.0 (8.0, 13.0)	9.0 (6.0, 13.0)	0.67	9.0 (5.5, 13.0)	0.36
SDI (score), median (range)	0.0 (0.0, 2.0)	0.0 (0.0, 1.0)	1.5 (0.0, 2.0)	0.5 (0.0, 2.0)	0.46	0.0 (0.0, 1.0)	0.64
**Cumulative organ involvement:**							
Mucocutaneous	33 (75%)	18 (90%)	7 (70%)	8 (57%)	0.07	60 (75%)	1.00
Rash	22 (50%)	11 (55%)	3 (30%)	8 (57%)	0.37	42 (53%)	0.85
Oral ulcers	10 (23%)	7 (35%)	2 (20%)	1 (7%)	0.21	20 (25%)	0.83
Cutaneous vasculitis	10 (23%)	5 (25%)	2 (20%)	3 (21%)	1.00	6 (8%)	**0.02**
Arthritis	23 (52%)	12 (60%)	4 (40%)	7 (50%)	0.59	45 (56%)	0.71
Serositis	13 (30%)	7 (35%)	4 (40%)	2 (14%)	0.34	13 (16%)	0.11
Cardiac involvement	14 (32%)	6 (30%)	4 (40%)	4 (29%)	0.84	10 (13%)	**0.02**
Valvular calcification	7 (16%)	1 (5%)	2 (20%)	4 (29%)	0.17	3 (4%)	**0.03**
PAH	5 (11%)	2 (10%)	3 (30%)	0 (0%)	0.10	3 (4%)	0.13
Coronary lesions	2 (5%)	1 (5%)	0 (0%)	1 (7%)	1.00	2 (3%)	0.61
Ascending aorta dilation	7 (16%)	4 (20%)	2 (20%)	1 (7%)	0.57	0 (0%)	**<0.01**
Heart failure	2 (5%)	1 (5%)	0 (0%)	1 (7%)	1.00	3 (4%)	1.00
Atrial fibrillation	3 (7%)	2 (10%)	1 (10%)	0 (0%)	0.59	3 (4%)	0.67
Nephritis	23 (52%)	9 (45%)	6 (60%)	8 (57%)	0.79	37 (46%)	0.58
Hematological involvement	31 (70%)	14 (70%)	7 (70%)	10 (71%)	1.00	54 (68%)	0.84
Venous thrombotic events	4 (9%)	1 (5%)	1 (10%)	2 (14%)	0.80	6 (8%)	0.74
**Comorbidities**							
Other autoimmune diseases	22 (50%)	10 (50%)	4 (40%)	8 (57%)	0.69	32 (40%)	0.34
pSS	8 (18%)	1 (5%)	3 (30%)	4 (29%)	0.10	21(26%)	0.38
Hashimoto thyroiditis	16 (36%)	9 (45%)	3 (30%)	4 (29%)	0.66	20 (25%)	0.22
**Traditional cardiovascular risk factors**							
Hypertension	20 (45%)	8 (40%)	7 (70%)	5 (36%)	0.27	29 (36%)	0.34
Diabetes	6 (14%)	3 (15%)	2 (20%)	1 (7%)	0.74	9 (11%)	0.78
Hyperlipidemia	17 (39%)	6 (30%)	4 (40%)	7 (50%)	0.48	29 (36%)	0.85
Atherosclerosis	5 (11%)	2 (10%)	2 (20%)	1 (7%)	0.69	9 (11%)	1.00
Smoking	6 (14%)	4 (20%)	0 (0%)	2 (14%)	0.45	6 (8%)	0.34
BMI (kg/m^2^, mean ± SD)	23.8 (22.0, 25.4)	24.1 (22.3, 25.8)	23.5 (21.4, 25.8)	23.4 (22.7, 24.8)	0.88	23.1 (20.3, 25.7)	0.17
**Immunologic indices**							
Anti-dsDNA	24 (55%)	13 (65%)	4 (40%)	7 (50%)	0.36	32 (40%)	0.13
Anti-Sm	11 (25%)	5 (25%)	1 (10%)	5 (36%)	0.41	25 (31%)	0.54
Anti-Ro/SSA	20 (45%)	11 (55%)	4 (40%)	5 (36%)	0.54	43 (54%)	0.45
Anti-La/SSB	5 (11%)	3 (15%)	2 (20%)	0 (0%)	0.22	11 (14%)	0.79
Anti-RNP	21 (48%)	12 (60%)	3 (30%)	6 (43%)	0.32	35 (44%)	0.90
Anti-ribosomal P	15 (34%)	9 (45%)	2 (20%)	4 (29%)	0.39	23 (29%)	0.55
aPLs							
aCL	15 (34%)	5 (25%)	4 (40%)	6 (43%)	0.56	9 (11%)	**<0.01**
Anti-β_2-_GPI	14 (32%)	3 (15%)	5 (50%)	6 (43%)	0.09	13 (16%)	0.07
LA	11 (25%)	4 (20%)	2 (20%)	5 (36%)	0.66	4 (5%)	**<0.01**
Low C3 and/or C4	36 (82%)	19 (95%)	6 (60%)	11 (79%)	**0.05**	67 (84%)	0.81
**Treatments:**							
GC	23 (52%)	15 (75%)	2 (20%)	6 (43%)	**0.01**	56 (70%)	**0.05**
median(range)	1.8 (0.0, 16.9)	9.3 (0.9, 22.1)	0.0 (0.0, 0.0)	0.0 (0.0, 15.3)	**0.03**	5.9 (0.0, 14.5)	0.36
HCQ	17 (39%)	11 (55%)	1 (10%)	5 (36%)	**0.05**	54 (68%)	**<0.01**
ISDs	19 (43%)	10 (50%)	2 (20%)	7 (50%)	0.26	51 (64%)	**0.04**
CYC	12 (27%)	6 (30%)	1 (10%)	5 (36%)	0.42	22 (28%)	1.00
MMF	3 (7%)	2 (10%)	1 (10%)	0 (0%)	0.59	15 (19%)	0.11
**SLE recurrence**	23 (52%)	13 (65%)	4 (40%)	6 (43%)	0.34	38 (48%)	0.81
Number of relapses, median (range)	1.0 (0.0, 2.0)	2.0 (0.0, 3.0)	0.0 (0.0, 2.0)	0.0 (0.0, 2.0)	0.18	0.0 (0.0, 1.0)	0.08
Causes of recurrence							
GC withdrawal	12 (27%)	8 (40%)	2 (20%)	2 (14%)	0.28	18 (23%)	0.66
GC reduction	15 (34%)	9 (45%)	2 (20%)	4 (29%)	0.39	19 (24%)	0.29
No/inadequate use of ISDs	17 (39%)	9 (45%)	4 (40%)	4 (29%)	0.67	16 (20%)	**0.03**

Data were presented as n (%), unless otherwise indicated.

ICVD, ischemic cerebrovascular disease; SLE, systemic lupus erythematosus; ICAS, intracranial arterial stenosis; IQR, interquartile range; SLEDAI-2K, the Systemic Lupus Erythematosus Disease Activity Index 2000; SDI, Systemic International Collaborating Clinics/American College of Rheumatology Damage Index; PAH, pulmonary arterial hypertension; BMI, body mass index; anti-dsDNA, anti-double stand DNA antibody; aPLs, antiphospholipid antibodies; aCL, anticardiolipin antibody; anti-β_2_GPI, anti-beta-2-glycoprotein I antibodies; LA, lupus anticoagulant; GC, glucocorticoid; HCQ, hydroxychloroquine; ISDs, immunosuppressive drugs; CYC, cyclophosphamide; MMF, mycophenolate mofetil.

^*^The follow-up duration was defined as the time from SLE onset to the last follow-up (non-ICVD-SLE) or to the first occurrence of ICVD (ICVD-SLE).

^†^The diagnostic delay was defined as the time from SLE onset to the diagnosis of SLE.

^‡^The P-values referred to the difference between ICVD-SLE group and non-ICVD-SLE group. The bolded texts refer to P-values lower than 0.05.

^§^The P_0_-values referred to the comparison among subgroups in ICVD-SLE group. The bolded texts refer toP_0_-values lower than 0.05.

### Traditional cardiovascular risk factors

We compared the traditional cardiovascular risk factors between patients in the ICVD-SLE group and non-ICVD-SLE group (**Table 1**). No statistically significant differences between the groups were found in coexisting comorbidities (hypertension, diabetes, hyperlipidemia, atherosclerosis, ever-smoking, or body mass index (BMI)).

### Clinical manifestations, laboratory indices, and treatments

Among all the clinical manifestations, there were significant differences in the frequency of cutaneous vasculitis (23% vs. 8%, *P*=0.02) and cardiac involvement (32% vs. 13%, *P*=0.02) including valvular calcification (16% vs. 4%, *P*=0.03) and ascending aorta dilatation (16% vs. 0%, *P*<0.01) between patients in the ICVD-SLE group and the control group. The positivity rates for aCL and LA were elevated (34% vs. 11%, *P*<0.01 and 25% vs. 5%, *P*<0.01, respectively), while the prevalence of anti-β2-GPI antibody positivity increased in the ICVD-SLE group (32% vs. 16%, *P*=0.07) when compared to the control. There was no significant difference between groups in SLEDAI scores at SLE onset [10.0 (7.0–13.0) vs. 9.0 (5.5–13.0), *P*=0.36], SDI scores at the occurrence of ICVD or at the date of the latest medical record [0.0 (0.0–2.0) vs. 0.0 (0.0–1.0), *P*=0.64), positivity of other autoantibodies, or the rate of hypocomplementemia [36 (82%) vs. 67 (84%), *P*=0.81] (**Table 1**).

Of the 44 patients with ICVD, 20 (45%) had large-vessel ischemic stroke, 22 (50%) had symptomatic lacunar stroke, 4 (9%) had TIA, 2 (4.5%) had both large-vessel ischemic stroke and symptomatic lacunar stroke, 12 (27%) had subcortical infarction, while 9 (20%) had cortical infarction. Stroke was the first clinical manifestation for 3 (6.8%) patients. Some of the patients not only suffered from ICVD, but also had other neuropsychiatric manifestations, with 11 (25%) having mood disorders and anxiety, 9 (20%) having headaches, 8 (18%) having cognitive dysfunction, 6 (14%) having epileptic seizures, 5 (11%) psychosis, and 3 (7%) in an acute confusional state (ACS). The median mRS at 90 days after the occurrence of ICVD was 2.5 (1.2–3.7) (**Table 2**).

**Table 2:** The characteristics of neurological involvement in 44 lupus patients with ischemic cerebrovascular disease.

	Total N=44	No ICAS N_1_ = 20	Moderate ICAS N_2_ = 10	Severe ICAS N_3_ = 14	$P_{0}^{§}$
**Other NP events:**					
Headache	9 (20%)	4 (20%)	2 (20%)	3 (21%)	1.00
ACS	3 (7%)	2 (10%)	0 (0%)	1 (7%)	0.79
Psychosis	5 (11%)	4 (20%)	0 (0%)	1 (7%)	0.28
Epilepsy	6 (14%)	2 (10%)	1 (10%)	3 (21%)	0.64
Post-stroke epilepsy	4 (9%)	1 (5%)	0 (0%)	3 (21%)	0.19
Cognition	8 (18%)	3 (15%)	0 (0%)	5 (36%)	0.09
Mood disorders	11 (25%)	5 (25%)	1 (10%)	4 (29%)	0.59
Depression	8 (18%)	3 (15%)	1 (10%)	4 (29%)	0.53
mRS, median (range)	2.5 (1.2, 3.7)	2.0 (1.0, 4.0)	1.0 (0.0, 3.0)	3.0 (2.0, 4.0)	0.14
**Brain regions involved:**					
Frontal	30 (68%)	15 (75%)	8 (80%)	7 (50%)	0.23
Temporal	14 (32%)	4 (20%)	2 (20%)	8 (57%)	0.06
Parietal	21 (48%)	9 (45%)	5 (50%)	7 (50%)	1.00
Occipital	13 (29%)	3 (15%)	1 (10%)	9 (64%)	**<0.01**
Insular	7 (16%)	4 (20%)	0 (0%)	3 (21%)	0.28
Centrum semiovale	9 (20%)	3 (15%)	1 (10%)	5 (36%)	0.31
Corona radiata	10 (23%)	4 (20%)	1 (10%)	5 (36%)	0.38
Paraventricular	14 (32%)	7 (35%)	4 (40%)	3 (21%)	0.65
Basal ganglia	14 (32%)	7 (35%)	2 (20%)	5 (36%)	0.77
Cerebellar	8 (18%)	2 (10%)	2 (20%)	4 (29%)	0.41
Brain stem	7 (16%)	1 (5%)	2 (20%)	4 (29%)	0.17
Large-vessel ischemic infarction	20 (45%)	8 (40%)	4 (36%)	8 (62%)	0.44
Cortical infarction	9 (20%)	2 (10%)	2 (20%)	5 (36%)	0.22
Subcortical infarction	12 (27%)	7 (35%)	4 (40%)	1 (7%)	0.12
TIA	4 (9%)	3 (15%)	0 (0%)	1 (7%)	0.54
Hemorrhagic stroke	2 (45%)	0 (0%)	0 (0%)	2 (14%)	0.14
Cerebral small-vessel disease	31 (70%)	14 (74%)	9 (90%)	8 (57%)	0.24
CMBs	2 (4%)	1 (5%)	0 (0%)	1 (7%)	1.00
WMHs	22 (50%)	9 (45%)	6 (60%)	7 (50%)	0.80
Total Fazekas score ≥2–3^Δ^	15 (34%)	5 (25%)	5 (50%)	5 (36%)	0.69
Fazekas score, median (range)	2.0 (1.0–3.0)	1.0 (1.0–3.0)	2.0 (2.0–3.0)	2.0 (1.0–3.0)	0.32
Lacunar infarcts	22 (50%)	8 (40%)	6 (60%)	8 (57%)	0.58
Atrophy	11 (25%)	6 (30%)	3 (30%)	2 (14%)	0.61
ICVD recurrence	21 (48%)	10 (50%)	2 (20%)	9 (64%)	0.12
Time interval between relapses (months), median (range)	12.0 (3.0–33.0)	6.0 (1.0, 24.0)	5.0 (0.0, 36.0)	18.0 (2.0, 30.0)	0.76

Data were presented as n (%), unless otherwise indicated.

NP, neuropsychiatric; ACS, acute confusional state; mRS, modified Rankin scale; TIAs, transient ischemic attacks; WMHs, white matter hyperintensities.

^§^The P_0_-values referred to the comparison among subgroups in ICVD-SLE group. The bolded texts refer to P_0_-values lower than 0.05.

^Δ^Total Fazekas score ≥3 referred to periventricular WMH Fazekas ≥3 (extending into the deep white matter) and/or deep WMH Fazekas ≥2–3 (confluent or early confluent) (5).

Among the 124 patients, more than 50% of patients received glucocorticoids (79/124, 63.7%), immunosuppressive drugs (ISDs, 70/124, 56.4%), and HCQ (71/124, 57.3%). The most frequently used immunosuppressant was cyclophosphamide (CYC, 34/124, 27.4%), followed by mycophenolate mofetil (MMF, 18/124, 14.5%). No significant difference was observed between groups in the median cumulative dosages of glucocorticoids [1.8 (0.0–16.90 vs. 5.9 (0.0–14.5), *P*=0.36]. However, the usage rates of glucocorticoids, ISDs, and HCQ were significantly insufficient in patients in the ICVD-SLE group when compared to the control (52% vs. 70%, *P*=0.05; 43% vs. 64%, *P*=0.04; 39% vs. 68%, *P*<0.01, respectively) (**Table 1**).

After the onset of SLE, all the patients were followed up on for 10–108 months (median 48 months). During the follow-up period, 49.2% of the lupus patients had relapsed. There was no significant difference between groups in the recurrence rate of SLE. Although the median number of SLE recurrence showed no significant difference between groups, it indicated a trend toward an increase in the ICVD-SLE group [1 (0–2) vs. 0 (0–1), *P*=0.08], with the prevalence of inadequate use of immunosuppressants significantly higher in the ICVD-SLE group (39% vs. 20%, *P*=0.03), among the common causes for disease recurrence. In the ICVD-SLE group, 48% of patients had two or more ICVD events, and the median time interval between the events was 12 (3–33) months (**Table 1**).

### Brain MRI of patients with ischemic cerebrovascular disease

All the 44 patients in the ICVD-SLE group and 23 (28.75%) patients in the non-ICVD-SLE group had brain MRI. In the ICVD-SLE group, cerebral infarcts were mainly located in the frontal lobe (30/44, 68%), followed by the parietal lobe (21/44, 48%), the temporal lobe (14/44, 32%), the basal ganglia region (14/44, 32%), the periventricular region (14/44, 32%), and the occipital lobe (13/44, 29%). Multiple infarcts in the brain were found in 88.6% of the ICVD-SLE patients. The brain MRI characteristics of cerebral SVDs were found in 70% of the patients in this group, the most common features of which were lacunar infarcts (22/44, 50%) and WMHs (22/44, 50%) with 34% (15/44) having total Fazekas ≥3, followed by brain atrophy (11/44, 25%) and CMBs (2/44, 4%) (**Table 2**).

### Vascular ultrasonography imaging of patients with ischemic cerebrovascular disease

In the ICVD-SLE group, the median number of intracranial arteries involved for each patient was 1 (0–1). Concurrent extracranial cerebral arteries were involved in 62% (27/44) of the patients. Anterior cerebral artery (ACA) was the most common intracranial large artery involved (32%), followed by the vertebrobasilar artery (VBA) (23%). Of these patients, 25% (10/44) had moderate ICAS, 6.8% (4/44) had severe ICAS, and 23% (10/44) had total occlusion (**Table 3**). Carotid intimal thickening and plaques were found in 45% (20/44) of the patients (**Table 3**).

**Table 3:** The findings of cerebral arterial Doppler ultrasonography scanning in 44 lupus patients with ischemic cerebrovascular disease.

	Total N=44	No-ICAS N_1_ = 20	Moderate ICASN_2_ = 10	Severe ICAS N_3_ = 14	$P_{0}^{§}$
Number of involved intracranial arteries, median (range)	1.0 (0.0–5.0)	0.0 (0.0, 0.0)	1.5 (0.0, 2.0)	1.0 (1.0, 3.0)	–
Type of involved intracranial arteries, median (range)	1.0 (0.0–3.0)	0.0 (0.0, 0.0)	1.5 (0.0, 2.0)	1.0 (1.0, 2.0)	–
Extracranial cerebral arteries involved^*^	27 (62%)	10 (63%)	10 (100%)	7 (50%)	**0.02**
ACA	14 (32%)	0 (0%)	5 (50%)	8 (57%)	–
MCA	5 (11%)	0 (0%)	2 (20%)	3 (21%)	–
PCA	5 (11%)	0 (0%)	2 (20%)	3 (21%)	–
VBA	10 (23%)	0 (0%)	5 (50%)	4 (29%)	–
Intracranial ICA	6 (14%)	0 (0%)	4 (40%)	2 (14%)	–
Carotid wall thickening	20 (45%)	8 (40%)	6 (60%)	6 (43%)	0.68
Carotid plaque	20 (45%)	6 (30%)	9 (90%)	5 (36%)	**<0.01**
Arterial occlusion	10 (23%)	0 (0%)	0 (0%)	10 (23%)	–

Data were presented as n (%), unless otherwise indicated.

ACA, anterior cerebral artery; MCA, middle cerebral artery; PCA, posterior cerebral artery; VBA, vertebrobasilar artery; ICA, internal carotid artery.

^§^The P_0_-values referred to the comparison among subgroups in the ICVD-SLE group. The bolded texts refer to P_0_-values lower than 0.05.

*Referred to the major branches of the aorta, including brachiocephalic trunk (BCT), subclavian artery (SCA), common carotid artery (CCA), extracranial internal carotid artery (ICA).

### Analysis of the risk factors for ischemic cerebrovascular disease in lupus patients

**Figure 1:**
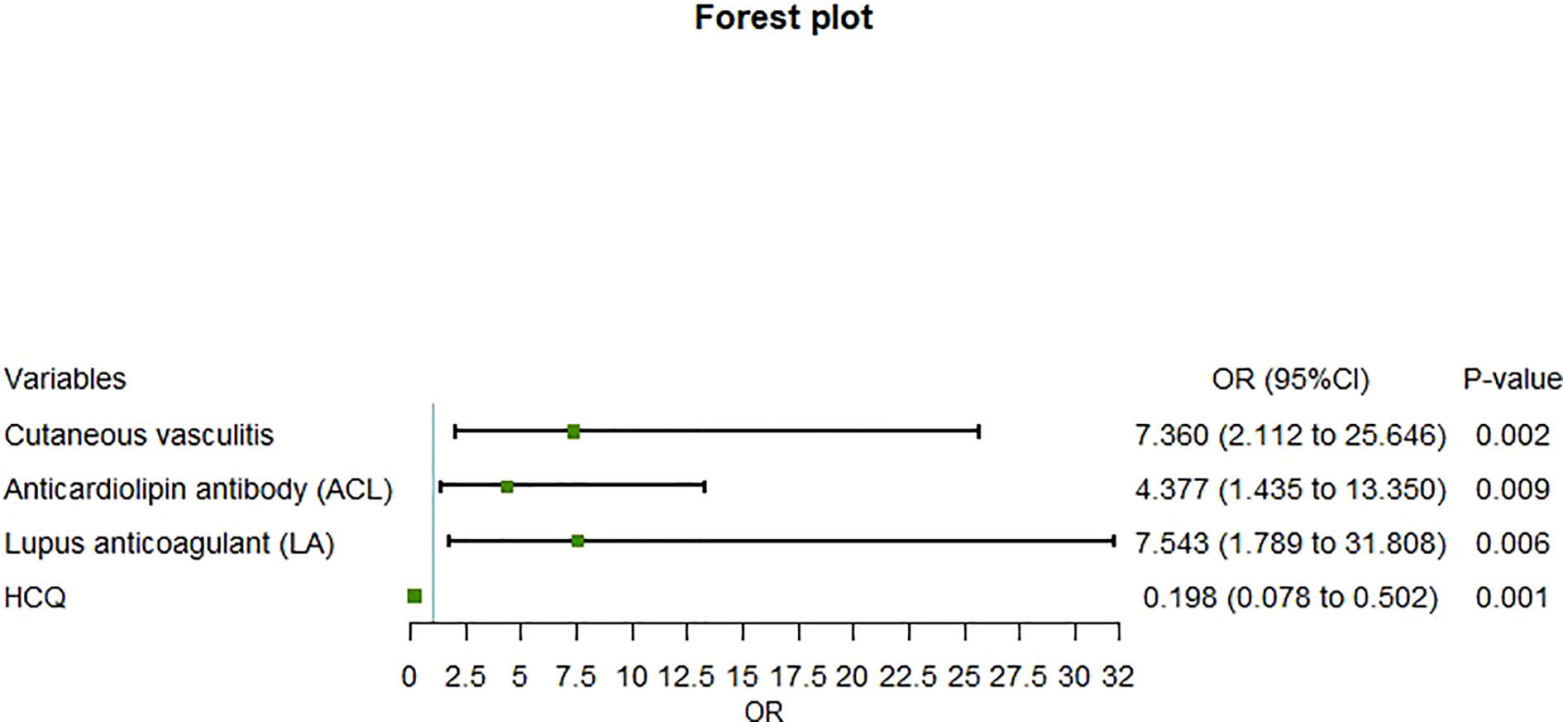
Forest plot of multivariate regression analysis of risk factors for ischemic cerebrovascular disease in patients with systemic lupus erythematosus.

Although age, gender, duration, BMI, and traditional cardiovascular risk factors were included in the logistic multivariate regression model because of their clinical relevance, their predictive power for ICVD was lost when cutaneous vasculitis, aCL, LA, and HCQ therapy were included in this model. It was indicated that cutaneous vasculitis (OR=7.36, 95% CI=2.11–25.65, *P*=0.002), aCL (OR=4.38, 95% CI=1.435–13.350, *P*=0.009), and LA (OR=7.543, 95% CI=1.789–31.808, *P* = 0.006) were independent risk factors for ICVD in lupus patients, while HCQ therapy (OR=0.198, 95% CI=0.078–0.502, *P*=0.001) was identified as an independent protective factor (**Figure 1**).

### Sub-analysis of the lupus patients with ischemic cerebrovascular disease, according to the gradations of intracranial arterial stenosis

In the ICVD-SLE group, 24 (24/44, 54.5%) patients had ICAS. According to the gradations of ICAS, all 44 patients in the ICVD-SLE group were divided into three subgroups: the no-ICAS group (n=20), the moderate ICAS group (n=10), and the severe ICAS group (n=14). **Tables 1**–**3** show the clinical manifestations, brain MRI imaging, and vascular US characteristics of the subgroups. Of note, no significant difference was observed between the patients of the no-ICAS group and the severe ICAS group, except that patients in the severe ICAS group had a trend of prolonged diagnostic delay of less than 1 year. However, patients in the moderate ICAS group were older at the onset of SLE [56.0 (40.0, 62.0) years, *P*<0.01], experienced a longer diagnostic delay [26.0 (8.0, 46.0) months, *P*<0.01], showed lower prevalence of hypocomplementemia (55%, n=6, *P*= 0.02), used less glucocorticoids (18%, n=2, *P*<0.01) and HCQ (9%, n=1, *P*=0.04), trended toward lower mRS scores at 90 days after the occurrence of ICVD [1.0 (0.0, 3.0), *P*=0.06], and trended toward decreased recurrence of ICVD but had higher rates of the presence of carotid plaque (82%, n=9, *P*=0.02) when compared to the other groups.

In patients in the severe ICAS group, there was a higher rate of prevalence of cognitive disorders (38%, n=5, *P*=0.05), and the cerebral infarcts were more often located in the temporal (62%, n=8, *P*=0.03) and occipital (69%, n=9, *P*<0.01) lobes when compared to the other groups. In addition, there was no significant difference among the subgroups in the prevalence of large-vessel ischemic stroke or cerebral SVD

## Discussion

This retrospective case-control study evaluated the risk factors of ICVD in patients with SLE based on our single-center data. It was first proved that cutaneous vasculitis, in addition to aPLs, as reported in previous research, significantly correlated with ICVD in patients with SLE as reported in previous research. In the subgroup analysis according to the gradations of ICAS, we found that ICVD in young and middle-aged patients with SLE was characterized by complement-mediated systemic inflammation and a relatively unfavorable outcome that required intensive immunosuppressive therapy. In elderly patients with SLE, ICVD was characterized by moderate ICAS and carotid atherosclerotic plaques.

### Mechanisms of ischemic cerebrovascular disease in systemic lupus erythematosus

As previously reported, atherothrombosis is the most common pathological process, accounting for about 15% of all ischemic strokes in population-based studies (17). In addition, increasing age, hypertension, diabetes, metabolic syndrome, and ever-smoking were also found as traditional cerebrovascular risk factors (6, 18, 19). Meanwhile, it is known that emboli that lead to ischemic stroke originate in the heart (most commonly due to atrial fibrillation and release of potentially harmful material from an abnormal valvular surface) and the aortic arch (due to aortic arch atheroma) or the large arteries (20). It is reported that aortic arch atheroma with presence of plaques ≥4-mm thick is a significant risk factor for new (21) and recurrent ischemic stroke (22, 23). However, lupus can cause endothelial activation and contribute to the development of atherosclerosis and thrombus formation, involving both large and small vessels, which may cause TIA or ischemic stroke. It can also involve the heart by forming non-infectious vegetations on the cardiac valves. This can result in embolic stroke or cause cardiac arrest or arrhythmia, cardiac output reduction secondary to myocardial infarction, or pericardial effusion, and lead to hypoperfusion stroke. Additionally, aPLs, which are present in 11%–40% of patients with SLE (24, 25) and 17.2% in young patients (<50 years) with a stroke (26), are considered a cause of an acquired hypercoagulable state which can lead to ischemic stroke and TIA. aPLs comprise a heterogeneous group of autoantibodies, including mainly anti-β_2_-glycoprotein I antibody (aβ_2_-GPI), aCL, LA, and other antiphospholipid–protein antibodies. Therefore, ICVD in SLE involves multifactorial etiology with inflammatory burden and traditional cardiovascular risk factors (27, 28).

### Antiphospholipid antibodies and ischemic cerebrovascular disease in systemic lupus erythematosus

In our present study, we did not find a significant difference in the prevalence of hypertension, diabetes, hyperlipidemia, ever-smoking, or obesity (BMI) among groups. This might be partially attributed to the relatively small sample size of the study. However, the significant increase in incidence of stroke and TIA was still seen in individuals younger than 55 years without these risk factors, as Li et al. reported, which supported our findings (29, 30). Moreover, in our study, we found that the prevalence of aPLs in lupus patients with ICVD was 25%–34%, and the incidence of aPLs positivity, especially aCL and LA positivity, was significantly higher versus patients in the non-ICVD group. aCL and LA were demonstrated to be the independent risk factors for ICVD in patients with SLE, which was in accordance with previous studies (31–34). Demir. (35) demonstrated that in SLE, LA was the best predictor of venous and arterial thrombosis, and especially in arterial thrombosis, no other antiphospholipid antibody (including IgA aβ_2_-GPI antibody) had an additive risk to LA. Other prior studies indicated that antiphospholipid antibody thrombosis in lupus patients was considered more severe in association with the presence of LA and with a persistently positive aCL level (36, 37). A large multicenter population-based case–control study (32) confirmed that LA was a major risk factor, not only in SLE, but also for arterial thrombotic events in young women; this risk further increased with the existence of other cardiovascular risk factors. Reynaud et al. (38) demonstrated by a meta-analysis that LA and aCL had the highest OR for arterial thrombosis compared to the other aPLs in adults without SLE. Wan et al. indicated that LA positivity was an independent associated factor with brain MRI abnormalities (especially lacunae and WMHs) in people without neuropsychiatric symptoms (39), However, in our study, we tested lupus patients with IgG/IgM aβ_2_-GPI (targeting β_2_-GPI Domain I) and IgG/IgM aCL and did not further investigate IgA aβ_2_-GPI (targeting β_2_-GPI Domain III IV V) or identify β_2_-GPI-dependent aCL IgG. Their diagnostic and clinical significance have been well demonstrated in other studies, which indicates they are correlated with arterial thrombosis (26, 40, 41). This issue will be further explored in our future research.

### Cutaneous small-vessel vasculitis and ischemic cerebrovascular disease in systemic lupus erythematosus

Cutaneous vasculitis (CV) is a non-specific cutaneous presentation with the highest incidence among the various types of vasculitis in patients with SLE. It mainly exhibits as punctate lesions, palpable purpura, ulcers, erythematous plaques or macules, and erythema with necrosis (42–44). It is reported to be associated with systemic vasculitis (including lupus nephritis), increased organ damage, and hypocomplementemia (45, 46). CV, as a small-vessel vasculitis, mediated by circulating immune complexes (ICs) or by directly binding antibodies to cellular superficial components. ICs are formed in the microvasculature, and contribute to complement-mediated inflammation, and are frequently deposited on the basement membranes of skin (47). In this study, we indicated that CV is an independent risk factor for ICVD in patients with SLE. This finding is supported by previous studies. Callen and Kingman reported that CV was correlated with disease activity and poor prognosis with renal involvement and central nervous system (CNS) deterioration (48). A retrospective study of juvenile Asian patients with SLE indicated an increased risk of renal and neuropsychiatric manifestations in patients with CV versus a control group (49). Patients with cutaneous vasculitis were more likely to have anti-ribosomal P antibodies (50), anti-Ro antibody (51), and cryoglobulins (52), which were all considered to be strongly associated with CNS involvement in SLE (53–57). However, two cross-sectional studies from Brazil that investigated both adult (50) and juvenile patients with SLE showed that all clinical manifestations, including CNS involvements, showed no significant difference between SLE patients with or without CV (58). Gomes et al. confirmed that digital vasculitis was not associated with severe lupus manifestations, particularly renal and CNS involvements (59). These results might be because the researchers excluded patients with antiphospholipid syndrome (APS), which might have influenced the result, since antiphospholipid antibodies and vasculopathy were thought to play an important role in the mechanism of CV in lupus patients (42, 43). However, it is worth noting that non-vasculitis occlusive vasculopathy might be similar to vasculitis lesions (60). Vasculitis, as an inflammatory process, engages infiltration of the vessel walls by leucocytes with subsequent endothelial injury and fibrinoid necrosis (61). Vasculopathy, as a non-inflammatory lesion, results from coagulopathy (correlated with aPLs) that leads to occlusion of dermal blood vessels with fibrin thrombi (62). Since skin biopsies are not required in clinical practice, patients in our cohort did not have a skin biopsy; thus, CV was identified based on clinical manifestations alone (63). Therefore, our findings needed to be validated and further refined in future research.

### Hydroxychloroquine and ischemic cerebrovascular disease in systemic lupus erythematosus

In this study, we also found that HCQ was an independent protective factor against ICVD in lupus patients, which was consistent with several previous studies. A nested case–control study within inception cohorts of SLE and RA patients, including the entire population of British Columbia, Canada, found a statistical trend towards reductions in stroke, which suggested a possible cardiovascular preventative effect of HCQ (64). Petri et al. examined HCQ blood levels in a longitudinal SLE cohort to prove that low HCQ blood levels were associated with thrombotic events in SLE (65). It was indicated that HCQ could block intracellular calcium signaling in macrophages and lymphocytes (66, 67) and attenuate anti-ribosomal P-induced neurotoxicity by preventing intracellular calcium signals and apoptosis in neurons (68). HCQ could also prevent β2-glycoprotein I complex from binding to phospholipid bilayers and cells and protect the annexin A5 anticoagulant shield from being destroyed by antiphospholipid antibodies, which was considered to decrease the risk of thrombosis in APS and SLE (69, 70). As a result, as Petri et al. proved, HCQ should be used with personalized dosage, which cannot be easily reduced empirically in this high-risk population if there are no contraindications.

### Subgroup analysis of lupus patients with ischemic cerebrovascular disease

The subgroup analysis of the lupus patients with ICVD revealed interesting findings that patients in the moderate ICAS group were significantly older (>50 years) at disease onset, had prolonged diagnostic delay, high prevalence of atherosclerotic carotid plaques, and fewer immunosuppressive therapies undertaken before the occurrence of ICVD. On the other hand, patients in the other two groups showed no significant difference in group comparisons, except that patients in the severe ICAS group had their diagnosis delayed for nearly 1 year and used less immunosuppressive agents before the occurrence of ICVD than those in the no-stenosis group. They presented convergent features, such as being younger than 50 years at disease onset, prevalence of hypocomplementemia, and a relatively unfavorable outcome. It is possible that the severe stenosis or even occlusion of the intracranial arteries in these younger lupus patients was due to a diagnostic delay and lack of immunosuppressive therapy. These findings strongly indicate that inflammation in this population is lupus-related and requires timely and adequate immunosuppressive therapy (71). That means attribution of ICVD to SLE is still a challenge because, in this case, immunosuppressive treatment is urgently needed because of its protective role in disease control and reduction of disease recurrence (72). However, as other researchers have demonstrated, strokes attributed to SLE are inclined to occur within 1 year around disease diagnosis and may be due to systemic inflammation, endothelial activation, or a prothrombotic state due to aPLs (10). In contrast, strokes unrelated to SLE usually occur at late stages and are induced by atherosclerosis resulting from traditional cardiovascular risk factors, which represent common comorbidities in SLE (73). In our study, we used two different parameters, the “diagnostic delay” and the “follow-up duration,” which was the time interval from disease onset to the occurrence of the first ICVD event. Thus, we found that patients in the no-ICAS group and severe ICAS group were considered to have ICVD attributed to SLE, which occurred nearly 5–8 years after disease onset, with a diagnostic delay of less than 1 year. It means that ICVD attributed to SLE in our cohort occurred around 4–7 years after diagnosis of SLE. It was contradictory to the findings that strokes attributed to SLE usually occur close to diagnosis. Again, our study confirmed that patients in the moderate ICAS group were indicated to have ICVD more likely attributed to atherosclerosis, which occurred less than 2 years after disease onset and close to diagnosis. This was also not comparable with previous conclusions that strokes unrelated to SLE occur at late stages. Conversely, we hypothesized that elderly lupus patients with ICVD presented with less lupus-related inflammation, but accelerated atherosclerosis induced by inflammation (74–76). Therefore, these patients might have relative atypical symptoms, a non-rapidly progressive course, and easily be delayed for diagnosis. Our findings should be further validated. There may be different phenotypes in SLE with different susceptibilities to ICVD based on genomics, epigenomics, and transcriptomics.

### Hypocomplementemia and ischemic cerebrovascular disease in systemic lupus erythematosus

We also found that hypocomplementemia might be an indication of systemic inflammation associated with ICVD attributed to SLE. Abnormalities in the complement cascade and immune complex-mediated complement consumption played a protagonist role in the pathogenesis of SLE and disease activity. Prior published studies confirmed that persistent hypocomplementemia in the first year after diagnosis was considered the only serologic marker of poor prognosis (39). It was indicated that the complement cascade was also activated in antiphospholipid-related thrombosis (77, 78). *In vitro* studies demonstrated that complement deposition on platelets in lupus, irrespective of the presence of antiphospholipid antibodies (79), was associated with venous (80) and in other research, arterial thrombosis (81). Cohen et al., in their post-mortem histopathological study of the brains of NPSLE and SLE patients, found that complement deposition might play a central role in the interaction between autoantibodies and thrombo-ischemic lesions observed in Neuropsychiatric lupus (NPSLE) (82). The presence of cell-bound complement activation products and the presence of the LA and low C3 were indicated to predict thrombosis in SLE (83). Moreover, Durcan and Petri. have shown that the combination of low C3 and low C4, accompanied with antiphospholipid antibodies, was associated with stroke (84). In their other research, they demonstrated that hypocomplementemia may represent an additional risk factor for vascular events in the presence of aPLs (85). Therefore, our findings are generally in accordance with evidence from previous research and need further in-depth studies in order to accurately identify better treatments for this unique population.

### Characteristics of the location of ischemic lesions

Based on the findings of MRI and vascular Doppler US, we concluded that in lupus patients in the severe ICAS group, ACA and VBA were most commonly involved, with occipital and temporal lobes being the most common brain regions in which the infarcts were localized. Additionally, these patients had higher rates of cognitive disorder when compared to the control group. It is well known that luminal narrowing or even occlusion can induce distal progressive ischemia or infarction, which is associated with cognitive decline (86). However, although ACA was the most common intracranial large artery involved in our cohort, the stroke territories were mainly in the posterior circulation instead of the anterior one, which might indicate that the anterior and posterior circulations have different vulnerabilities to abnormal blood supply, or that there are different mechanisms for stenosis of ACA and VBA in SLE patients. Notably, as is well acknowledged, in reversible posterior encephalopathy syndrome (PRES), which is commonly reported in SLE (87), the posterior circulation is particularly intolerant of fluctuations in blood pressure (BP) due to its relatively lower sympathetic innervation (88). In addition, the pro-inflammatory and cytotoxic environment found in SLE patients could result in microcirculatory dysfunction, increased plasma leakage, and brain hypoperfusion (89, 90). These hypotheses for PRES may explain our findings to some extent in this study that ischemic lesions are prone to locate in the posterior circulation territories; this requires further research.

### Increased small-vessel disease burden

In our study, 70% of the 44 patients with ICVD had imaging evidence of SVD, with lacunar infarcts (50%) and WMHs (50%) the most common features, given that the average age of these patients was younger than 40 years. This might be another piece of evidence of the lupus-mediated inflammation involving the brain, which is in accordance with previously reported literature (7, 91). Although most of the lupus patients in the non-ICVD group did not have MRI scans, multiple previous studies have reported that even in SLE patients without neuropsychiatric manifestations, there is increased WMH lesion load (92–94), which has been demonstrated to be closely associated with cerebral infarcts, aPLs, and high general SLE activity, along with traditional factors such as age and hypertension (92). Moreover, several prospective MRI studies have indicated that a higher load of deep WMH lesions would progress over time and were independently associated with new stroke onset (95, 96). Thus, we will further investigate SVD in SLE in future studies.

### Limitations

Some limitations of our study should be mentioned. This was a single-center, retrospective study, limited by the single reference center recruitment, the small sample size, relatively wide confidence intervals, and restricted ethnicity. The results might not be directly applicable to other ethnic populations. Moreover, an in-depth subgroup analysis for exploration of risk factors could not be adequately carried out due to the small sample size in each subgroup. Therefore, it is necessary to expand the sample size in the later stages and conduct a multicenter prospective study for further validation. Additionally, no cognitive testing or depression/anxiety scales nor brain MRI scans and cerebral arterial Doppler US were conducted with a certain number of SLE patients in the non-ICVD group. Thus, we could not further evaluate the relationship of cognition and mood disorders with ICVD in SLE, nor could we investigate the parameters of brain MRI imaging and vascular Doppler US as potential biomarkers or risk factors for ICVD in SLE. Moreover, patients with undetected asymptomatic lacunar infarction in the non-ICVD-SLE group might cause a selection bias.

## Conclusions

In conclusion, in our retrospective single-center case-control study, we found that the presence of cutaneous vasculitis and aPLs were risk factors for ICVD in SLE, while receiving HCQ therapy was a protective factor against ICVD in SLE. In the subgroup analysis, on the basis of the gradations of ICAS, we found that younger ICVD patients with SLE were associated with complement-mediated systemic inflammation and poor outcomes and required timely and intensive immunosuppressive therapy. On the other hand, elderly lupus patients with ICVD were characterized by moderate vascular stenosis and carotid atherosclerotic plaques. If these findings are verified through further research, they could potentially provide stratified therapeutic guidance to diverse populations of people with lupus.

## Data availability statement

The original contributions presented in the study are included in the article/Supplementary Material. Further inquiries can be directed to the corresponding author.

## Ethics statement

This study was reviewed and approved by the Ethics Committee of Xuanwu Hospital, Capital Medical University. The patients/participants provided their written informed consent to participate in this study.

## Author contributions

LS was responsible for the study design, patient recruitment, data acquisition, and article drafting. ZQ was responsible for the assessment of the brain MRI imaging. SG helped with the statistical analysis. LW helped with data acquisition. YZ was responsible for the study design, article review, and final approval of this article. All authors contributed to the article and approved the submitted version.

## Funding

This work was supported by the National Natural Science Foundation of China (Grant/Award Number: 82001733).

## Acknowledgments

We thank Fei Zhao and Chunxiu Wang for critical comments regarding the statistical analysis in this study.

## Conflict of interest

The authors declare that the research was conducted in the absence of any commercial or financial relationships that could be construed as a potential conflict of interest.

## Publisher's note

All claims expressed in this article are solely those of the authors and do not necessarily represent those of their affiliated organizations, or those of the publisher, the editors and the reviewers. Any product that may be evaluated in this article, or claim that may be made by its manufacturer, is not guaranteed or endorsed by the publisher.
